# Glucose Metabolites Exert Opposing Roles in Tumor Chemoresistance

**DOI:** 10.3389/fonc.2019.01282

**Published:** 2019-11-21

**Authors:** Chung-Yen Huang, Ching-Ying Huang, Yu-Chen Pai, Been-Ren Lin, Tsung-Chun Lee, Pi-Hui Liang, Linda Chia-Hui Yu

**Affiliations:** ^1^Graduate Institute of Physiology, National Taiwan University College of Medicine, Taipei, Taiwan; ^2^School of Medicine, National Taiwan University College of Medicine, Taipei, Taiwan; ^3^Department of Food Science and Biotechnology, National Chung-Hsing University, Taichung City, Taiwan; ^4^Department of Surgery, National Taiwan University Hospital and College of Medicine, Taipei, Taiwan; ^5^Department of Internal Medicine, National Taiwan University Hospital and College of Medicine, Taipei, Taiwan; ^6^School of Pharmacy, National Taiwan University, Taipei, Taiwan

**Keywords:** colorectal carcinoma, chemotherapy resistance, glycolytic pyruvate, liposomal ATP, reactive oxidative species, necroptotic death, cell cycle progression

## Abstract

Reprogrammed glucose metabolism and increased glycolysis have been implicated in tumor chemoresistance. The aim was to investigate the distinct roles of the glucose metabolites pyruvate and ATP in chemoresistance mechanisms, including cell death and proliferation. Our data showed higher glucose transporters in colorectal cancer (CRC) from non-responsive patients than those responsive to chemotherapy. Human CRC cell lines exposed to 5-fluorouracil (5-FU) displayed elevated cell viability and larger tumors in xenograft mouse models if cultured in high-glucose medium. Glucose conferred resistance to 5-FU-induced necroptosis via pyruvate scavenging of mitochondrial free radicals, whereas ATP replenishment had no effect on cell death. Glucose attenuated the 5-FU-induced G0/G1 shift but not the S phase arrest. Opposing effects were observed by glucose metabolites; ATP increased while pyruvate decreased the G0/G1 shift. Lastly, 5-FU-induced tumor spheroid destruction was prevented by glucose and pyruvate, but not by ATP. Our finding argues against ATP as the main effector for glucose-mediated chemoresistance and supports a key role of glycolytic pyruvate as an antioxidant for dual modes of action: necroptosis reduction and a cell cycle shift to a quiescent state.

## Introduction

Colorectal cancer (CRC) is the second leading cause of cancer-related mortality. Resistance to chemotherapies such as first-line genotoxic agents represents a significant problem in the clinical management of CRC. Recurrent cancers often become more aggressive than the original tumors due to subclonal selection by the cytotoxic pressure (1). Early studies on tumor chemoresistance have focused on genetic changes in association with drug metabolism and efflux pathways (2, 3). Accumulating evidence suggests that altered cellular bioenergetic status, which contributes to cancer growth, is also involved in chemoresistance (4, 5).

Glucose metabolic reprogramming is a well-known phenomenon to fuel tumor growth (6, 7). Upregulation of glycolytic enzymes and abnormal expression of specific glucose transporters (GLUT-1 to 4) and sodium glucose transporter 1 (SGLT1) were associated with enhanced proliferation and metastasis of human CRC (8–10). Recurrence of CRC after preoperative chemotherapy was correlated with high GLUT1 expression and anaerobic glycolysis in tumors (11–13). Recent evidence showed that hyperglycemia and high sugar content in the intestinal lumen enhanced tumor growth and compromised treatment effectiveness in mouse models (14, 15). Increased risk of recurrence and mortality of CRC has also been related to high dietary glycemic load, fasting hyperglycemia, and diabetes (16–18). Malabsorption and colonic retention of dietary sugars were found in CRC patients undergoing chemotherapy (19, 20), indicating that high glucose supply from both blood circulation and colonic lumen may promote CRC growth and chemoresistance. To date, the concept of glycolytic energy demand has been extrapolated to justify the metabolic chemoresistance mechanisms, albeit without supporting data.

Intracellular glucose is normally metabolized by anaerobic glycolysis to pyruvate which entered the mitochondrial tricarboxylic acid (TCA) cycle to produce the majority of ATP to support cell division and many biological functions (21). Thus far, ATP yield has been generally considered the main effector for promoting chemoresistance and drug efflux via ATP-binding cassette transporters in tumor cells (5, 22, 23). Nevertheless, contradictory data have cast doubt on the regulatory role of ATP, whereby the administration of exogenous ATP augmented the chemosensitivity of tumor cells by accelerating cell proliferation and genotoxicity (24, 25).

Excessive nutrient uptake results in incomplete catabolism and excretion of intermediates in conditions of hyperactive cellular metabolism such as cancers (26, 27). In the Warburg effect, which is characterized by increased glucose uptake and a preference of glycolysis even in an aerated environment, the glycolysis rate is much faster (~100 times) than the rate for complete mitochondrial oxidation of glucose and the excess carbon from glycolysis is converted to lactate (28). Unconventional fuel sources converted by pyruvate, such as lactate and acetate, enabled tumors to meet metabolic demands in an harsh environment with nutrient scarcity such as in the core of a poorly vascularized tumor mass (29). A recent study demonstrated that pyruvate was converted to acetate through coupling to reactive oxygen species (ROS), of which acetate served as an alternative carbon source for metabolism (30). Our laboratory and others demonstrated that pyruvate, by scavenging ROS, rescued cells from hypoxia-induced cell death (31–33). Furthermore, accumulating evidence indicated that subtoxic levels of mitochondrial ROS were essential for cancer cell proliferation (34–36). Therefore, we suspected that glycolytic pyruvate may act as a free radical scavenger and modulate the tumor response to chemotherapy. Taken together, the individual roles of the glucose metabolites pyruvate and ATP in tumor chemoresistance have yet to be explored.

5-Fluorouracil (5-FU) is the most commonly used genotoxic drug for treating CRC. As a uracil-mimicking agent, 5-FU exerts anti-tumor effects by inhibiting thymidylate synthase to block DNA synthesis and by causing S-phase arrest to halt cell proliferation (37, 38). 5-FU also triggers various types of cell death, including apoptosis, necrosis, and a novel form of programmed necrosis known as necroptosis (39, 40). Receptor-interacting protein (RIP) kinases and mitochondrial ROS generation are involved in the necroptotic machinery (41). Cancer cells escaped 5-FU-induced cell death by entering a quiescent state during cell cycle progression (42). Previous studies have demonstrated that high rates of glucose uptake caused a reduction in 5-FU-induced cytotoxicity (43, 44). Moreover, chemicals that modulate glucose metabolism, such as 3-bromopyruvate, iodoacetate, dichloroacetate, and 2-deoxyglucose, also sensitized cancer cells to 5-FU (45, 46). Here, we suspect that glucose metabolites (i.e., pyruvate and ATP) may play divergent roles in 5-FU chemoresistance relating to resistance to cell death and modulation of cell proliferation.

The overall aim of this work was to correlate glucose transporter expression in human CRC with chemotherapy outcomes and to investigate the molecular mechanisms of glucose-mediated 5-FU chemoresistance. Our results demonstrated that glucose metabolites conferred drug resistance through multiple mechanisms, including a novel role of glycolytic pyruvate in the suppression of 5-FU-induced cell death by free radical scavenging and in the promotion of drug insensitivity by shifting cells from a proliferative state to a quiescent state. Our data challenge the common belief that ATP is the main effector of glucose-mediated chemoresistance and support a key role for pyruvate in promoting tumor cell survival by modulating cell death and cell cycle progression.

## Materials and Methods

### Surgical Specimens From Colorectal Cancer Patients

Colorectal cancer (CRC) patients undergoing adjuvant chemotherapy after surgery at National Taiwan University Hospital (NTUH) were recruited as subjects. Frozen samples of surgical specimens from age- and tumor stage-matched patients who were responsive (*N* = 14) or non-responsive (*N* = 11) to adjuvant chemotherapy regimens containing high dose 5-fluorouracil (5-FU) were assessed (Supplementary Table 1). Written informed consent was obtained from all study subjects, and approval for this study was granted by the Research Ethics Committee of NTUH (200912049R).

### Quantitative Polymerase Chain Reaction (qPCR)

Total RNA was extracted from samples using Trizol reagent (Life Technologies). The RNA (2 μg) was reverse transcribed with random primers using RevertAid™ First Strant cDNA Synthesis kit (Thermo) in 20 μL reaction volume. The resulting cDNA corresponding to 50 ng of initial RNA was then subjected to qPCR by the addition of Power SYBR Green Master Mix (Thermo) containing SYBR Green I Dye, AmpliTaq Gold DNA Polymerase, dNTPs, and ROX passive reference dye, 125 nM upstream primer, and 125 nM downstream primer.

The StepOne Real-Time PCR System (Thermo) was programmed to perform a protocol as follows: 95°C for 10 min for 1 cycle, followed by 95°C for 15 s (denaturation) and 60°C for 1 min (annealing and extension) for 40 cycles. Melting curve analysis confirmed the specificity of the qPCR reaction. Quantification was performed by calculating the comparative Ct values, normalizing the target gene expression to the reference gene (β-actin).

### Animal Models of CRC

Mice were subjected to protocols for chemical induction of CRC by administration of azoxymethane (AOM) and dextran sodium sulfate (DSS) as previously described (47, 48). All animal experiments were approved and monitored by the Laboratory Animal Care Committee in National Taiwan University College of Medicine.

### Xenograft Mouse Models

The human colorectal adenocarcinoma cell line HT29 (ATCC#HTB-38) was injected into immunodeficient mice to create a xenograft tumor model. The HT29 cells were suspended in a 1:3 ratio of Matrigel (Corning #354248) to culture medium containing normal glucose (5 mM) or high glucose (25 mM), and either plated *in vitro* for image analysis on spheroid growth (see below), or subcutaneously injected to mice *in vivo*. Immunodeficient NOD.CB17-Prkdc(scid)/JNarl mice (NOD/SCID) were used as the recipients to allow xenograft tumor growth. HT29 cells (2 × 10^6^) in a 200-μl volume of Matrigel-medium containing saline vehicle and either normal or high glucose were subcutaneously injected into the right and left flanks of each mouse, respectively. In another mouse group, HT29 cells suspended in the Matrigel-medium containing 5-FU (0.02 mM) and either normal or high glucose were injected in the same manner. The experimental design of injecting cells incubated in media containing saline or 5-FU into two separate mouse groups instead of injecting those on both flanks of mice was to avoid unwanted permeation of the anticancer drugs into blood circulation which may confound the data of saline group. Moreover, paired injection of normal and high glucose-containing media to the flanks of each mouse may circumvent differences in transplant rejection among subjects. The tumor size and body weight were monitored every 2–3 days until the tumor volume reached 2,500 mm^3^ as the end point. The tumors were resected and fixed in 4% paraformaldehyde for tissue embedding followed by histological sectioning to confirm the dysplastic morphology.

### Cell Culture Models

The human colorectal carcinoma cell lines HT29, HCT116, SW480, and Caco-2 were grown in Dulbecco's Modified Eagle's Medium (DMEM) containing 5 mM glucose and no pyruvate (Life Technologies, Carlsbad, CA) (32, 47). Cells were exposed to various doses of 5-fluorouracil (5-FU) for 48 h in culture medium containing 1, 5, or 25 mM glucose (32, 47). In other experiments, cells were challenged with 5-FU in the presence of equimolar concentrations of a cell-permeable pyruvate derivative, ethyl pyruvate, in glucose- and pyruvate-free culture media (32, 33). Moreover, ATP-encapsulated and empty liposomes at 0–1,000 μM were added to 5-FU-treated cells in glucose- and pyruvate-free culture medium (33). The liposomes were provided by Dr. Chin-Tin Chen from the Department of Biochemical Science and Technology, National Taiwan University.

The cell viability and cell death levels were measured by using commercialized kits. The cell cycle progression and cell proliferation were assessed by flow cytometric analysis and quantification of spheroid growth (see below).

### Cell Viability Tests

The cell viability was determined by using a tetrazolium dye MTT (3-(4,5-dimethylthiazol-2-yl)-2,5-diphenyltetrazolium bromide) assay (Cayman Chemical, Ann Arbor, MI) according to the manufacturer's instruction. The MTT assay is a colorimetric assay for measuring the metabolic activity of NAD(P)H-dependent cellular oxidoreductase enzymes which reflect the number of viable cells present. The half maximum inhibitory concentration (IC50) of 5-FU was calculated using a GraphPad Prism software (GraphPad Software Inc., CA, USA).

### Measurement of Cell Death Levels

Apoptosis characterized by DNA fragmentation was measured by a Cell Death Detection ELISA kit (Roche, Basel, Switzerland) according to the manufacturer's instruction (47). Necrosis/Necroptosis was measured by lactodehydrogenase (LDH) leakage assay as described (32, 33).

### Immunoprecipitation of RIP1-RIP3 Complex

Cells lysates were immunoprecipitated with anti-human RIP1 (BD bioscience) overnight, and then incubated with protein G agarose beads for 1 h at 4°C followed by centrifugation as described (32, 33). The pellet was dissolved in electrophoresis sample buffer for heat denaturation. The immune complexes were subjected to reducing SDS/PAGE and the membranes were incubated with anti-RIP1 (1:1,000, BD bioscience) or polyclonal rabbit anti-RIP3 (1:1,000, Abcam, Cambridge, UK) for immunoblotting and resolved by SDS/PAGE.

### Evaluation of Cell Cycle Progression

Cells treated with 5-FU were harvested for cell cycle analysis by flow cytometry. After the challenge, the cells were washed with PBS and fixed with ice-cold 70% ethanol for 24 h. The cells were incubated with anti-Ki67 antibody (1:500, LSBio) for 1 h, followed with goat anti-mouse IgG conjugated to Alexa Fluor^®^ 488 fluorescent probe (1:1,000, Molecular Probes). The cells were then incubated with propidium iodide (PI) for 30 min at room temperature. A minimum of 10,000 PI-stained nuclei were analyzed by flow cytometry, and the percentage of cells in the G0, G1, S, and G2–M phases of the cell cycle was determined using FlowJo cell cycle analysis software (FlowJo LLC, Oregon, USA) (47, 48).

### Spheroid Cultures

Cells were plated as three-dimensional spheroid cultures based on previous protocols (47, 48) with some modifications. The monolayered cells were trypsinized and resuspended in DMEM containing 25 mM glucose. The cells were then mixed with ice-cold Matrigel (Corning #354234) and cell culture medium at a 3:1 ratio, and 300 μl of the suspension was seeded per well in 24-well-plates. Spheroids were cultured for 4 days and exposed to 5-FU for 48 h. Twenty-four hours prior to 5-FU addition, the overlaying medium was changed to DMEM containing 5 mM glucose. The spheroids were then treated with 5-FU by replacing the overlaying medium with cell culture medium (900 μl) containing various doses of glucose and either pyruvate or liposomal ATP.

The spheroid size and structure before and after 5-FU exposure were observed, and the microscopic images were captured using a CCD camera, and analyzed using ImageJ software 1.47v. The percentage of spheroids with structural dissociation was determined. Destructed spheroids were defined as lacking a circular shape and displaying indentation and notches on the spheroid surface. A total number of 100–120 spheroids were quantified per experimental group.

### Measurement of Mitochondrial Free Radicals

Mitochondrial-derived reactive oxidative species (ROS) generation was measured by using MitoSOX (Invitrogen). The cells were incubated with MitoSOX (5 μM) for 20 min after 5-FU treatment, and then subjected to fluoremetric readings (32).

Details regarding the measurement of intracellular ATP, pyruvate, lactate, and acetate contents; immunofluorescence staining; Western blotting; and statistical analysis are included in Supplementary Materials and Methods.

## Results

### Higher Glucose Transporter Expression in Colorectal Cancers of Non-responsive Patients Than in Patients Responsive to Chemotherapy

**Figure 1:**
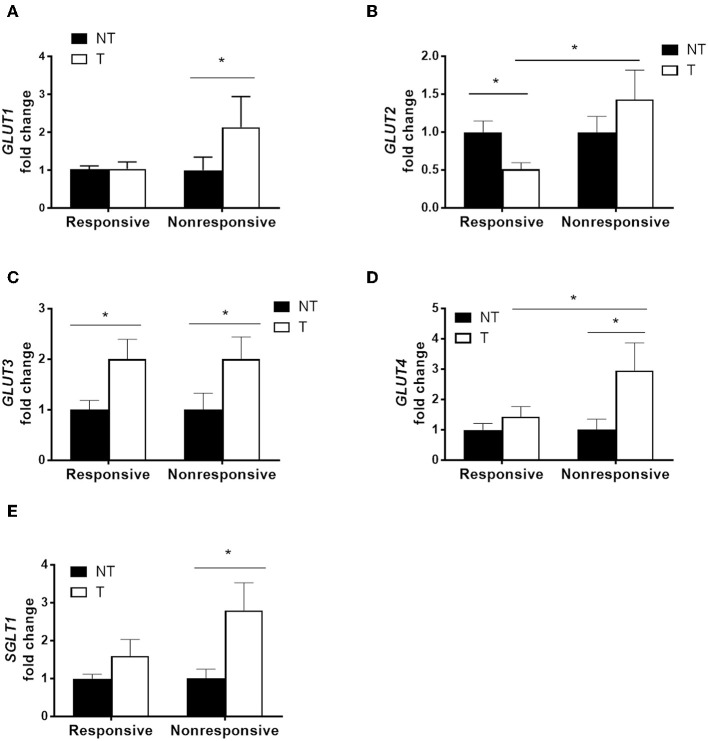
Higher glucose transporter expression in tumor specimens correlated with poor chemotherapy outcome in patients with colorectal cancers. Paired tumor (T) and adjacent non-tumor (NT) tissues were obtained from patients that underwent adjuvant chemotherapy containing high-dose 5-fluorouracil (5-FU). The patients were divided into two groups based on tumor recurrence [i.e., responsive (*N* = 14) and non-responsive (*N* = 11)]. **(A–E)** Transcript levels of glucose transporters (GLUT1, GLUT2, GLUT3, GLUT4, and SGLT1) were examined by real-time PCR. Paired samples *t*-tests were performed for NT vs. T; independent samples *t*-tests were performed for Responsive vs. Non-responsive. ^*^P < 0.05.

Cancer specimens from patients who received adjuvant chemotherapy containing high dose of 5-FU were divided into two groups based on tumor recurrence (Supplementary Table 1). The mRNA levels of glucose transporters in paired samples of tumor specimens and adjacent non-tumor tissues from each patient were quantified by real-time PCR. Significantly higher levels of *GLUT1, GLUT4*, and *SGLT1* transcripts were observed in the tumor tissues of non-responsive patients compared to those of responsive patients (Figures 1A,D,E). Increased levels of *GLUT3* transcript were found in the tumor tissues of both non-responders and responders (Figure 1C). Moreover, no difference in *GLUT2* was observed between the tumor and normal tissues in non-responders, whereas decreased *GLUT2* levels in tumors were noted in the responders (Figure 1B).

### Abnormally Located or Elevated Expression of Glucose Transporters in Mouse Colorectal Cancers

**Figure 2:**
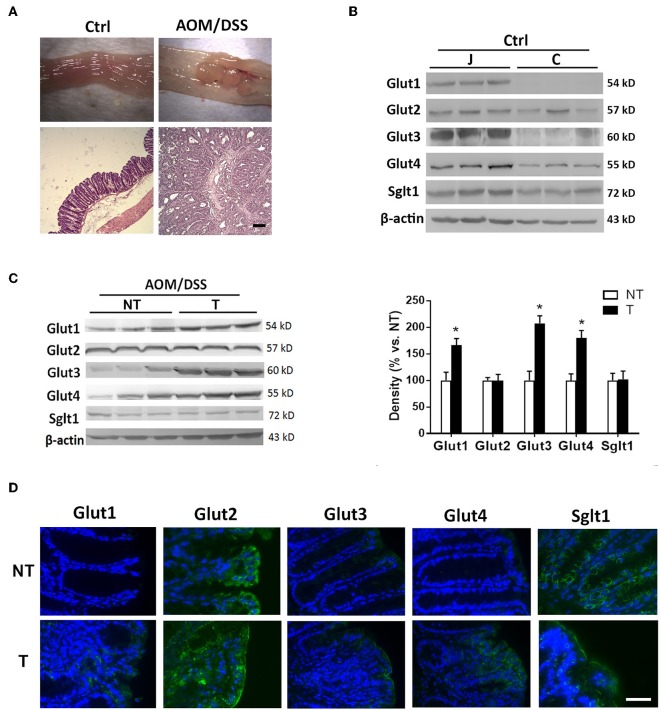
Glucose transporters were abnormally expressed or increased in mouse colon cancers. Mice were administered azoxymethane (AOM)/dextran sodium sulfate (DSS) to induce distal colon tumors. **(A)** Macroscopic and histological images of colonic tissues in untreated control (Ctrl) and AOM/DSS mice. Bar: 50 μm. **(B)** Expression of glucose transporters in mucosal tissues of jejunum (J) and colon (C) in Ctrl mice. **(C)** Expression of glucose transporters in non-tumor (NT) and tumor (T) tissues of AOM/DSS mice. **(D)** Representative immunofluorescent images showing expression patterns of glucose transporters in NT and T tissues of mice. Bar: 50 μm. Paired samples *t*-tests were performed. ^*^P < 0.05. *N* = 6/group.

Mice were administered carcinogens to develop tumors in the distal colon for evaluation of glucose transporters. Normal intestinal mucosa of untreated control mice as well as tumor and adjacent non-tumor tissues obtained from chemically induced CRC mice were assessed by Western blot (Figure 2A). The high levels of glucose transporters (Glut1-4 and Sglt1) in the normal jejunal mucosa served as positive controls for the proteins. Normal colonic mucosa showed low to negligible expression of glucose transporters (Figure 2B). The colon tumor samples showed elevated expression of Glut1, Glut3, and Glut4 compared to that in the adjacent non-tumor tissues (Figure 2C). No difference in Glut2 and Sglt1 levels was found between tumor and non-tumor tissues (Figure 2C).

Immunofluorescent staining revealed higher surface protein levels of Glut1, Glut3, and Glut4 in tumor samples than in adjacent non-tumor tissues (Figure 2D). Moreover, distinct subcellular localization of Glut2 and Sglt1 proteins was observed between non-tumor and tumor tissues despite no difference in the amount. Glut2 protein was found in the cytoplasm of non-tumor surface epithelial cells, whereas its expression was homogenously distributed in the tumor with expression at the apical membrane of surface cells (Figure 2D). In addition, the Sglt1 protein was located only in the crypts of non-tumor tissues with membranous expression, while an increase in luminal surface expression of Sglt1 was observed in tumors (Figure 2D). Overall, the data showed abnormal expression of glucose transporters in mouse tumors and elevated levels in human colorectal cancers that were recalcitrant to chemotherapy.

### Glucose Uptake Reduced Cytotoxicity of 5-FU in Cancer Cells

**Figure 3:**
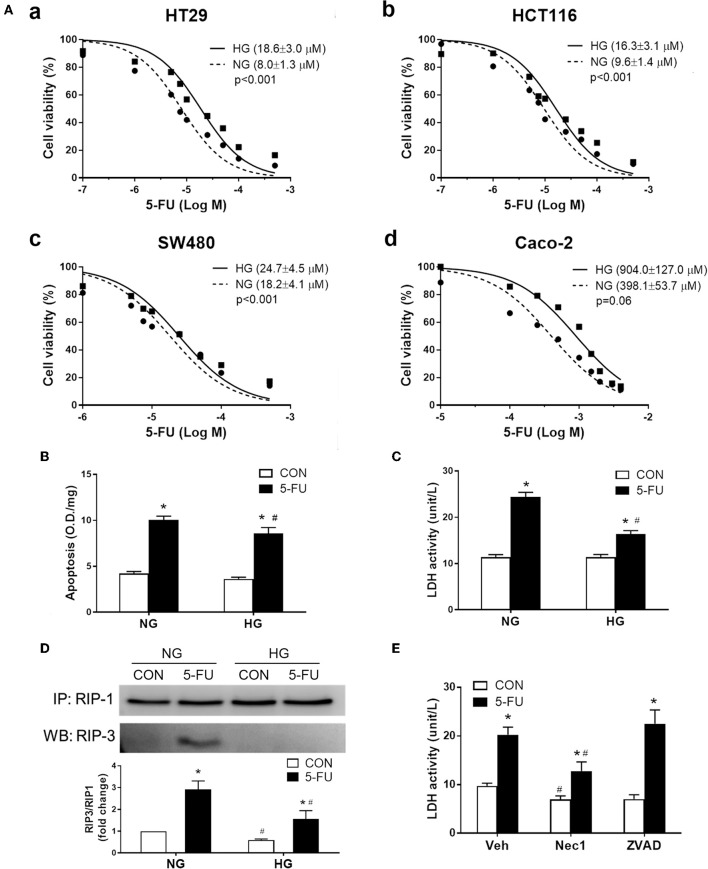
Human cancer cell death caused by 5-FU, including apoptosis and necroptosis, was partially inhibited by the presence of high glucose. **(A)** The cell viability of four human colorectal carcinoma cell lines **(a)** HT29, **(b)** HCT116, **(c)** SW480, and **(d)** Caco-2, after treatment with 5-FU at various doses for 48 h in cell medium containing normal glucose (NG, 5mM) or high glucose (HG, 25 mM) concentration. The brackets in each panel indicate the IC50 of 5-FU in the four cell lines under normal and high glucose. Extra-sum-of-squares *F*-tests were performed for IC50 comparison. **(B,C)** HT29 cells were exposed to IC50 of 5-FU (0.01 mM) under NG and HG for measurement of cell apoptosis by DNA fragmentation and cell necrosis by lactodehydrogenase (LDH) leakage. *N* = 6/group. ^*^P < 0.05 vs. respective CON; ^#^*P* < 0.05 vs. respective NG. **(D)** Western blots showing RIP1/3 complex formation by immunoprecipitation as an indicator of necroptosis. The relative densitometric values of RIP3 over RIP1 were shown. *N* = 4/group. **(E)** Pretreatment with necrostatin-1 (Nec-1, 100 μM; a specific RIP inhibitor) but not ZVAD-FMK (ZVAD, 120 μM; a pan-caspase inhibitor) blocked the 5-FU-induced LDH leakage under NG. *N* = 6/group. Experiments were repeated three times. Independent samples *t*-tests were performed. ^*^P < 0.05 vs. respective CON; ^#^*P* < 0.05 vs. respective Veh.

Given that increased glucose transporter expression was identified in the non-responsive tumor biopsies and mouse CRC, we examined whether high glucose uptake was involved in tumor chemoresistance using four human cancer cell lines. The high glucose concentration (25 mM) in culture media is known to increase glucose uptake in cells as evidenced by C^14^-labeled radioassays (49, 50) and may also represent conditions of hyperglycemia or colonic sugar retention in cancer patients. Higher cell viability under 5-FU treatment was observed in the presence of high glucose (25 mM) in human CRC cell lines, including HT29, HCT116, SW480, and Caco-2 cells (Figure 3A). The increase in glucose concentration caused a shift in the IC50 of 5-FU in the CRC cell lines with statistical significance (Figure 3A). Blockade of glucose uptake by phloretin (a GLUT inhibitor), but not phloridzin (an SGLT1 inhibitor), partly inhibited the IC50 shift in HT29 cells, suggesting that high glucose uptake was involved in resistance to 5-FU (Supplementary Figure 1A). In terms of the glucose transporter levels, the expression of GLUT1, GLUT2, GLUT4, and SGLT1 was unaltered after high glucose uptake in HT29 cells (Supplementary Figure 1B).

The following experiments assessing cell death and proliferative responses were designed to investigate the molecular mechanisms of glucose-mediated chemoresistance. The IC50 of 5-FU (0.01 mM) in HT29 cells was chosen for the mechanistic studies, which is compatible with the plasma concentration of 5-FU (7-14 μM) in CRC patients receiving continuous chemotherapy infusion over a 24-h period (51, 52). Cells exposed to 5-FU under normal glucose conditions displayed an increase in apoptosis and necrosis, as evidenced by DNA fragmentation and lactodehydrogenase (LDH) leakage, respectively (Figures 3B,C). In contrast, the presence of high glucose largely inhibited the necrosis-associated LDH activity whereas only a slight reduction of apoptotic DNA fragmentation was observed (Figures 3B,C). Because necrosis is regarded as an uncontrollable type of cell death, necroptosis characterized by receptor-interacting protein (RIP) kinase-dependent signals was verified by immunoprecipitation of RIP1/3 complex. Our results showed that 5-FU caused RIP1/3 complex formation, implicating the induction of necroptosis under normal glucose conditions (Figure 3D). The presence of high glucose reversed the necroptotic signal of RIP1/3 complex formation caused by 5-FU (Figure 3D). Moreover, pretreatment with necrostatin-1 (an RIP1 inhibitor) but not ZVAD-FMK (a pan-caspase inhibitor) also blocked 5-FU-induced LDH leakage (Figure 3E), confirming the induction of programmed necroptosis.

### Glycolytic Metabolism, but Not ATP, Rescued Cells From 5-FU-Induced Necroptosis

**Figure 4:**
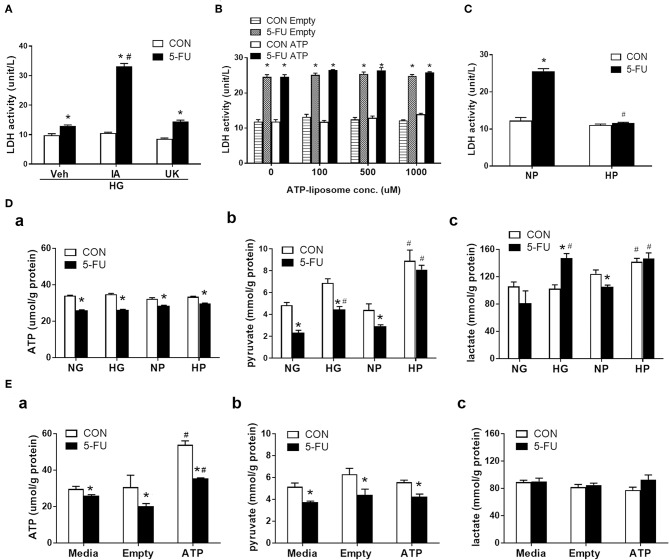
Glycolytic metabolism uncoupling to ATP was involved in the inhibition of 5-FU-induced necroptotic death. HT29 cells were exposed to 5-FU in the presence of glucose, liposomal ATP, or cell-permeable pyruvate. **(A)** High glucose (HG, 25 mM)-mediated inhibition of LDH leakage was prevented by iodoacetate (IA, 10 μM) (a glycolytic enzyme inhibitor) but not by UK5099 (UK, 10 μM) (a mitochondrial pyruvate carrier inhibitor). *N* = 6/group. ^*^P < 0.05 vs. respective CON; ^#^*P* < 0.05 vs. 5-FU Veh. **(B)** Addition of ATP-encapsulated liposomes did not reverse 5-FU-induced LDH leakage compared to those given empty liposomes in glucose- and pyruvate-free medium. *N* = 6/group. ^*^P < 0.05 vs. respective CON. **(C)** Presence of high pyruvate (HP, 25 mM) decreased the 5-FU-induced LDH leakage compared to those given normal pyruvate (NP, 5 mM) in glucose-free medium. A cell-permeable pyruvate derivative, ethyl pyruvate, was added to cells. *N* = 6/group. ^*^P < 0.05 vs. respective CON; ^#^*P* < 0.05 vs. 5-FU in NP. **(D)** The cellular levels of **(a)** ATP, **(b)** pyruvate, and **(c)** lactate in 5-FU-treated HT29 cells cultured in media containing glucose or pyruvate at normal (5 mM) and high (25 mM) concentrations. **(E)** The cellular levels of **(a)** ATP, **(b)** pyruvate, and **(c)** lactate in 5-FU-treated HT29 cells cultured in media containing empty and ATP liposomes (500 μM), or those without liposomes (Media). *N* = 4/group. Experiments were repeated three times. Independent samples *t*-tests were performed. ^*^P < 0.05 vs. respective CON; ^#^*P* < 0.05 vs. respective NG, NP, or Empty.

Intracellular glucose is normally metabolized by anaerobic glycolysis and oxidative mitochondrial respiration to produce ATP. In short, one glucose moiety is converted to two pyruvate molecules during glycolysis, which also generates two ATP molecules during the process. The pyruvate then enters the mitochondria through the mitochondrial pyruvate carrier (MPC) and serves as the starting substance in the TCA cycle to generate more energy (36 ATP). Here, the role of glucose metabolism as an energy source was investigated in the chemoresistance mechanism. Cells were pretreated with iodoacetate (IA, a glycolytic enzyme inhibitor) or UK5099 (UK, a MPC inhibitor) prior to 5-FU exposure under high glucose conditions. Pretreatment with IA but not UK prevented the glucose-mediated reduction in LDH leakage (Figure 4A), suggesting that glycolytic metabolism was involved in the resistance to 5-FU-mediated cell death.

Since both ATP and pyruvate were end products of glycolytic metabolism, their individual roles were evaluated in the chemoresistance mechanism. Liposomally encapsulated ATP was administered at various concentrations to 5-FU-treated cells cultured in glucose- and pyruvate-free media. However, none of the tested doses prevented 5-FU-induced LDH leakage (Figure 4B). In contrast, administration of cell-permeable pyruvate (at an equimolar concentration to glucose) reduced the levels of LDH leakage caused by 5-FU in HT29 cells (Figure 4C). The increase in cell viability and decrease of LDH leakage in the presence of high levels of pyruvate were confirmed in other human cell lines (Supplementary Figures 2A,B). Moreover, ATP had no effect on RIP signaling, while pyruvate reduced RIP1/3 complex formation caused by 5-FU in HT29 cells (Supplementary Figures 3A,B).

To evaluate glucose metabolism in 5-FU-treated cells, the intracellular levels of ATP, pyruvate, and lactate were assessed. HT29 cells exposed to 5-FU under normal glucose conditions displayed a drop in cellular ATP and pyruvate levels that correlated with cytotoxicity and cell death (Figures 4Da,b). The high glucose in culture media restored the cellular pyruvate content to baseline levels in the 5-FU-treated cells (Figure 4Db), confirming that high glucose uptake led to intracellular pyruvate synthesis. Nevertheless, the drop of ATP caused by 5-FU treatment was not reversed by high glucose (Figure 4Da).

The changes in glucose-related metabolites following administration of cell-permeable pyruvate and liposomal ATP were also verified. Similar to glucose, the addition of pyruvate in the culture media increased the intracellular pyruvate content but did not increase the ATP levels in 5-FU-treated cells (Figures 4Da,b). In contrast, liposomal ATP administration restored the levels of cellular ATP as expected (Figure 4Ea) and had no effect on the amount of cellular pyruvate (Figure 4Eb). Finally, increased lactate levels were only observed in groups cultured with medium containing high glucose or high pyruvate but not in those cultured with liposomal ATP (Figures 4Dc,Ec). The collective data with the pharmacological blockade results indicated that the high glucose taken up by 5-FU-treated cells, when rescued from cell death, underwent glycolysis for mainly pyruvate and lactate production without ending in the mitochondrial TCA cycles.

### Glycolytic Pyruvate Suppressed Mitochondrial Free Radical Production to Prevent Cell Death

**Figure 5:**
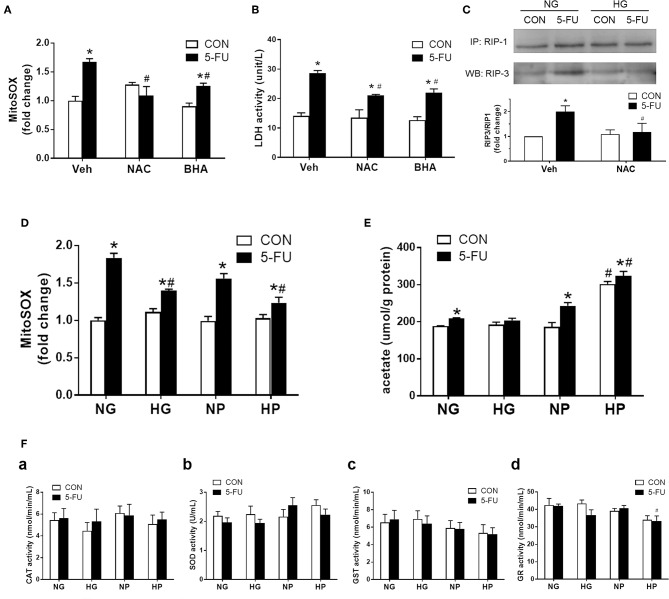
Glycolytic pyruvate suppressed mitochondrial free radicals which was involved in the necroptotic machinary. HT29 cells were exposed to 5-FU under normal glucose conditions, and the synthesis of mitochondrial free radicals were monitored. **(A)** The mitochondrial superoxide level was increased following 5-FU challenge under normal glucose, which was reduced by antioxidants including NAC (10 mM) or BHA (50 μM). **(B)** Pretreatment with antioxidants significantly decreased the 5-FU-induced cellular LDH leakage under normal glucose. *N* = 6/group. ^*^P < 0.05 vs. respective CON; ^#^*P* < 0.05 vs. 5-FU Veh. **(C)** Pretreatment with antioxidant NAC blocked the RIP1/3 complex formation caused by 5-FU. The relative densitometric values of RIP3 over RIP1 were shown. *N* = 4/group. **(D)** The 5-FU-induced mitochondrial superoxide synthesis was reduced by the presence of high glucose or high pyruvate. **(E)** The cellular acetate level was measured as a byproduct of pyruvate oxidation. High pyruvate led to increased intracellular acetate contents, indicating direct ROS scavening by pyruvate. **(F)** No increase in cellular redox enzyme activities were observed under high glucose or high pyruvate conditions. Activities of **(a)** catalase (CAT), **(b)** superoxide dismutase (SOD), **(c)** glutathione-S-transferase (GST), and **(d)** glutathione reductase (GR) were shown. *N* = 6/group. Experiments were repeated three times. Independent samples *t*-tests were performed. ^*^P < 0.05 vs. respective CON; ^#^*P* < 0.05 vs. respective NG, NP, or Empty.

Because glycolytic pyruvate uncoupling of mitochondrial transport or ATP replenishment was involved in glucose-mediated death resistance, we suspected that pyruvate as a free radical scavenger may play a role in the chemoprotective mechanism against necroptosis. Previous studies have shown that ROS was involved in the execution of necroptotic cell death caused by hypoxic and cytokine stress (32, 41). Here, mitochondrial superoxide production was induced by 5-FU treatment in HT29 cells under normal glucose conditions (Figure 5A). The reduction of superoxide levels by antioxidants, such as butylated hydroxyanisole (BHA) and N-acetylcysteine (NAC), decreased the 5-FU-induced cellular LDH leakage and RIP1/3 signaling (Figures 5A–C), supporting that ROS was involved in the necroptosis pathways caused by 5-FU. Moreover, high glucose and high pyruvate also significantly suppressed the mitochondrial superoxide levels in 5-FU-treated cells (Figure 5D and Supplementary Figure 3), suggesting that glucose metabolites were involved in the anti-death mechanisms through ROS scavenging.

A recent study demonstrated that pyruvate was oxidized to acetate through coupling to ROS in human colorectal HCT-116 cells (30). Therefore, the cellular acetate levels were measured in HT-29 cells as a byproduct for pyruvate oxidation. Elevated levels of acetate were seen in 5-FU-treated cells compared to untreated control cells in normal glucose conditions (Figure 5E), which correlated with the increased superoxide levels under genotoxic stress (Figure 5D). Moreover, a significant increase of acetate levels was observed in 5-FU-treated cells under high pyruvate (Figure 5E), further supporting that intracellular pyruvate oxidation to acetate was a means for free radical scavenging. In addition, there was no increase in the intracellular redox enzyme activities, such as catalase, superoxide dismutase, glutathione reductase or glutathione-S-transferase, by high glucose and pyruvate in 5-FU-treated cells (Figure 5F). Taken together, the findings supported an antioxidant role for pyruvate through direct coupling to free radicals, without modulating cellular redox activities.

### Distinct Effects of ATP and Pyruvate on Cell Proliferation of 5-FU-Treated Cells

**Figure 6:**
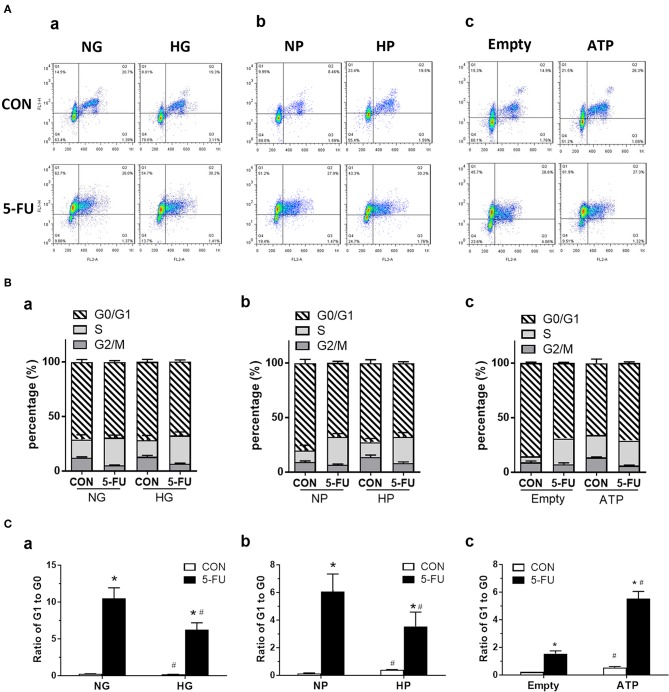
The 5-FU-induced G0/G1 shift of cell cycles was attenuated by glucose and pyruvate, but was aggravated by ATP. HT29 cells were exposed to 5-FU in the presence of glucose, pyruvate, or liposomal ATP. The cell cycles were analyzed by flow cytometry using staining of Ki67 (as FL1) and propidium iodide (PI) (as FL2). The PI binds to DNA by intercalating into nucleotide bases, and the intensity indicates the DNA contents during cell cycles of G1, S, G2, and M phases. In addition, actively proliferating cells express high Ki67 protein, whereas low Ki67 levels in cells indicate those in the quiescent phase of G0. **(A)** Dot plot of cell cycles, **(B)** Percentage (%) of cells in each phase, and **(C)** Ratio of cells in G1 to G0 phases, under high concentrations of **(a)** glucose, **(b)** pyruvate, or **(c)** ATP. *N* = 4/group. Experiments were repeated three times. Independent samples *t*-tests were performed. ^*^P < 0.05 vs. respective CON; ^#^*P* < 0.05 vs. respective NG, NP, or Empty.

Since 5-FU was originally design as a uracil-mimicking agent to halt tumor cell proliferation by incorporating to genomic DNA, the cell cycle distribution was next analyzed to examine how glucose and its metabolites modulate the proliferative responses of 5-FU-treated cells (Figure 6A). The cell cycle is characterized by four phases including the gap phase (G1 stage), DNA synthesis (S stage), growth phase (G2 stage), and mitosis phase (M stage). The G0 phase indicates the resting quiescence state where the cell has left the cycle and has stopped dividing. The cell cycles were analyzed by flow cytometry using staining of Ki67 and propidium iodide (PI). The PI binds to DNA by intercalating into nucleotide bases, and the intensity indicates the DNA contents during cell cycle progression. In addition, actively proliferating cells express high Ki67 levels, whereas cells in the G0 phase show low Ki67 levels. Our results demonstrated that the 5-FU treatment under normal glucose conditions resulted in a higher percentage of cells in the S phase and a higher ratio of G1 to G0, indicating that S phase arrest was triggered by genotoxic stress accompanied by a G0/G1 shift of the cells (Figures 6B,C). As recent reports showed that subtoxic levels of ROS were involved in cancer cell proliferation (34–36), we next verified whether free radicals played a role in the 5-FU-induced G0/G1 shift in cells. Pretreatment with NAC (a free radical scavenger) decreased the G0/G1 shift caused by 5-FU (Supplementary Figure 4), suggesting that pyruvate as a free radical scavenger may also regulate cell cycle progression.

In the presence of high glucose or high pyruvate, the 5-FU-induced G0/G1 shift was attenuated while no change in the S phase arrest was seen in HT29 cells (Figures 6B,C). In contrast, liposomal ATP aggravated the 5-FU-induced G0/G1 shift but also had no effect on S phase arrest (Figures 6B,C). The attenuation of 5-FU-induced G0/G1 shift by high pyruvate was confirmed in other human CRC cell lines (Supplementary Figure 5). Collectively, the data suggested that glycolytic pyruvate caused 5-FU insensitivity in tumor cells by promoting a cell cycle shift to a quiescence state and by reducing the proportion of actively proliferating cells that were able to take up the DNA-mimicking drug.

In untreated control cells, both cell-permeable pyruvate and liposomal ATP caused a G0/G1 shift in the cell cycle (Figures 6A–C), confirming that glycolytic metabolites and energy supply enhanced the proliferation of cells in a steady state.

### Glucose Promoted Tumor Chemoresistance in Xenograft Mouse Models

**Figure 7:**
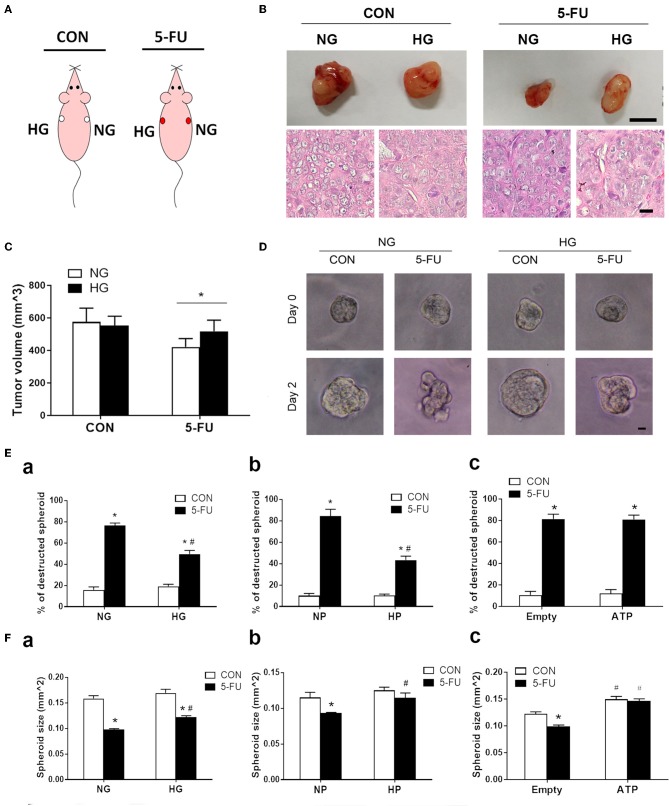
Glucose-mediated chemoresistance was observed in xenograft mouse models and tumor spheroid cultures. **(A)** Schema of the experimental design for xenograft models of immunodeficient mice which were divided into two groups: saline control (CON) and 5-FU. Each mouse was subcutaneously injected with HT29 cells in normal glucose (NG) and high glucose (HG) into the right and left flanks, respectively. **(B)** Representative photographs of the excised tumors and histological images were shown for each group. Upper bar: 10 mm. Lower bar: 10 μm. **(C)** Tumor size under normal or high glucose conditions in xenograft models. *N* = 6/group. Paired samples *t*-tests were performed. ^*^P < 0.05. **(D)** Representative images of HT29 spheroid cultures before (day 0) and after (day 2) exposure of 5-FU under normal and high glucose conditions *in vitro*. Bar: 10 μm. **(E)** The percentage (%) of destructed spheroids after 5-FU challenge under normal and high concentrations of **(a)** glucose, **(b)** pyruvate, or **(c)** liposomal ATP. **(F)** The spheroid sizes after 5-FU challenge under normal and high concentrations of **(a)** glucose, **(b)** pyruvate, or **(c)** ATP. Independent samples *t*-tests were performed. *N* = 6/group. ^*^P < 0.05 vs. respective CON; ^#^*P* < 0.05 vs. respective NG, NP, or Empty.

Finally, a xenograft mouse model was utilized to assess the effect of high glucose uptake on tumor chemoresistance *in vivo*. This model was designed to circumvent the drawback of fluctuating concentrations of blood glucose following meal ingestion and vascular transport. The mice were divided into two groups: saline control and 5-FU treatment. HT29 cells cultured in media with normal or high glucose were injected into the right and left flanks of each mouse, respectively (Figure 7A). The tumor sizes were examined on day 22 post-injection, and larger tumors were found in 5-FU-treated cells cultured in high glucose medium compared to those cultured in normal glucose medium (Figures 7B,C). No difference in tumor size was observed between untreated control cells cultured in normal and high glucose media (Figures 7B,C).

To correlate the xenograft tumor size with the tumor structural changes, three-dimensional spheroid cultures of HT29 cells were treated with 5-FU under high glucose, pyruvate or liposomal ATP for image analysis (Figure 7D and Supplementary Figure 6). Insufficient anticancer drug delivery to the core of a solid tumor results in inadequate cytotoxicity. The destruction and deformation of tumor mass could facilitate the rise of local concentrations of chemotherapeutic agents. We observed that 5-FU caused spheroid destruction and reduced the spheroid size in the presence of normal glucose, while high glucose partially attenuated these changes (Figures 7Ea,Fa). The effect of pyruvate on tumor structures was similar to that of glucose (Figures 7Eb,Fb). In contrast, liposomal ATP did not prevent the deformation of the spheroid mass (Figure 7Ec) but increased the spheroid sizes (Figure 7Fc). Together with the results in cell viability and cell cycle progression, the data suggested that glucose and pyruvate prevented the cell death-associated destruction in tumors caused by 5-FU, whereas ATP promoted tumor proliferation.

## Discussion

Tumor chemoresistance represents a significant problem in clinical management, and refractory tumors became more aggressive after first-line and targeted therapies (1). Early studies in chemoresistance have focused on heterogeneous changes associated with drug metabolism and efflux responses (2, 3). Here, we set a goal of identifying core protective pathways of chemoresistance regardless of the tumor heterogeneity. Our results showed for the first time the distinct modes of action through which glucose metabolites ATP and pyruvate fueled tumor chemoresistance by manipulating the balance between cell death and proliferation.

In the current study, a positive correlation was found between high expression levels of glucose transporters (i.e., GLUT1, GLUT3, GLUT4, and SGLT1) and refractory to adjuvant FOLFOX chemotherapy in CRC patients. Previous *in vitro* models of drug-resistant human CRC cell lines exhibited increased glycolysis compared to the susceptible parent cells (53, 54), supporting a link between heightened glycolytic metabolism and tumor chemoresistance. In mouse tumors, abnormally expressed glucose transporters with altered subcellular localization were found when comparing to normal intestinal epithelial cells. It is noteworthy that in human tumor specimens, specific transporters are aberrantly displayed (not merely increased) to meet the metabolic demand. Human enterocytes, the main entity involved in dietary sugar absorption, express SGLT-1 on the apical membrane and GLUT-2 for basolateral transport and utilize dietary glucose and glutamine as the primary energy source (49). Normal human colonocytes constitutively express GLUT-5 and−6 but lack other types of glucose transporters (55); short chain fatty acids provided half of the energy output in colonocytes, even in the presence of glucose (56). Our study provided evidence that aberrant transport and metabolism of glucose was correlated to chemoresistance in CRC. Further investigation is warranted to understand whether the relative contribution of serosal or luminal routes of glucose delivery may affect tumor responsiveness to chemotherapy. Overall, our data supported the notion that elevated glucose transport may drive tumor growth and chemoresistance.

All four human CRC cell lines (e.g., HT29, HCT116, SW480, and Caco-2 cells) showed higher cell viability with increasing glucose concentration when treated with 5-FU. Based on previous studies, these cell lines showed variable mutations in oncogenes (e.g., KRAS, β-catenin) and/or tumor suppressor genes (e.g., APC, DCC, and p53) (57, 58). The data demonstrated that glucose-mediated chemoresistance to 5-FU might be a universal phenomenon that is present in heterogeneous tumor cell lines that harbor different genetic mutations.

We assessed the modes of cell death and cell cycle transition by 5-FU treatment under normal glucose (5 mM, equivalent to 100 mg/dL blood glucose levels) and high glucose (25 mM). The high glucose concentration in culture media was correlated with increased glucose uptake in cells as previously shown by radioassay (49, 50) and also represented conditions of hyperglycemia or colonic sugar retention. A higher risk of CRC recurrence was linked to diabetes (16–18), whereby patients if not treated properly could display high levels of blood glucose (10–30 mM). Given that colonic retention of dietary sugars due to enteric malabsorption was observed in cancer patients (19), the strategic location of colonic tumors which allows for multiple routes of glucose delivery may compromise treatment effectiveness.

Different modes of cell death, including apoptosis, necroptosis, and necrosis, have been documented in cancer cell lines after treatment with 5-FU (39, 40, 59). Our results demonstrated that glucose uptake inhibited 5-FU-induced necroptosis but only showed slight effects on the rescue of apoptosis. Necroptosis is a newly identified form of cell death which is characterized by RIP kinase-dependent signals and synthesis of mitochondrial free radicals (32). The enhanced glucose uptake after 5-FU treatment prevented not only RIP1/3 complex formation but also ROS synthesis. Moreover, the anaerobic glycolytic pathway, but not mitochondrial respiration, was involved in the mechanism of glucose-mediated resistance to cell death, by which pyruvate recapitulated glucose protection in an ATP-independent manner. On the other hand, the restoration of cellular ATP levels did not reverse 5-FU-induced cell necroptosis, suggesting that energy was not the primary factor responsible for death resistance. Our data argue against the traditional view of ATP as the main chemoresistance factor by glucose metabolism and indicate a novel anti-necroptotic role of pyruvate under chemotherapy.

Because 5-FU was originally designed as a uracil-mimicking agent to curb cell proliferation by blocking DNA synthesis and causing S-phase arrest (37, 38), we assessed whether glucose uptake was capable of overcoming the arrest of cell cycle progression. Our results showed that neither glucose nor glycolytic metabolites (i.e., pyruvate and ATP) modulated 5-FU-induced S-phase arrest. Interestingly, a shift from G0 to G1 phase in the cell cycle was observed after 5-FU treatment. The G0/G1 shift signified that quiescent cells entered the division phase following 5-FU treatment, possibly as an compensatory response to maintain high levels of active proliferation—a hallmark characteristic of cancer. The phenomenon of G0/G1 shift after 5-FU treatment has not been previously reported and warrants further investigation. We showed that, similar to glucose, pyruvate triggered higher percentages of cells to enter a quiescent state. The finding is compatible with a report showing that ethyl pyruvate caused a blockade of tumor cell cycle progression at the G1 to S phase transition by inhibiting the extracellular release of a damage-associated molecular pattern protein (60). Recent studies indicated that subtoxic mitochondrial ROS production was essential for promoting cancer and epithelial cell proliferation (34, 35). Therefore, it is plausible that pyruvate-mediated free radical scavenging is involved in two fundamental cellular processes in stressed cells: death resistance and proliferation arrest. In contrast, ATP restoration aggravated the 5-FU-induced G0/G1 shift, suggesting that energy replenishment may be involved in tumor recurrence after the withdrawal of chemotherapy.

A growing body of evidence indicated that glycolytic end-product pyruvate acting as free radical scavengers played an important role in various biological functions. We demonstrated that pyruvate oxidation reduced the cellular ROS levels which led to decreased cell death and a cell cycle shift to a quiescent state, and hence, promoted the tumor cell survival during chemotherapy. Others documented that glycolytic pyruvate protected intestinal epithelial cells from hypoxia-induced apoptosis and necroptosis (32, 33). Recent studies showed that pyruvate converted to acetate via ROS coupling and to lactate via anaerobic pathways, both of which served as alternative mitochondrial carbon sources (29, 30). It remains unclear whether the cellular lactate and acetate entered the mitochondrial TCA cycles for energy production in the 5-FU-treated cells, which might be another means for tumor recurrence. In sum, glycolytic intermediate acted as a suppressor to oxidative stress and an alternative carbon resource that enabled tumor cells to survive and grow in harsh environments.

In conclusion, enhanced glucose uptake and aberrant glucose metabolism conferred 5-FU chemoresistance in colon cancer through distinct modes of cytoprotective mechanisms mediated by pyruvate and ATP. Glycolytic pyruvate provided resistance to cell death during chemotherapy, while ATP (as an energy source) promoted cell proliferation and may contribute to tumor recurrence after therapy withdrawal. A greater understanding of how glucose metabolites contribute to the fine tuning of cell fate decisions would benefit the development of interventions to overcome recalcitrant tumors in clinics.

## Data Availability Statement

The datasets generated for this study are available on request to the corresponding author.

## Ethics Statement

The studies involving human participants were reviewed and approved by Research Ethics Committee, National Taiwan University Hospital. The patients/participants provided their written informed consent to participate in this study. This animal study was reviewed and approved by Laboratory Animal Care Committee, National Taiwan University College of Medicine.

## Author Contributions

LY: guarantor of integrity of entire study concepts and design. Chu-YH, Chi-YH, and Y-CP: data acquisition. Chu-YH, Chi-YH, B-RL, T-CL, and LY: data analysis and interpretation. Chu-YH: statistical analysis. B-RL, T-CL, and P-HL: material and technical support. LY: obtained funding. All authors: manuscript drafting or revision for important intellectual content, literature research, manuscript editing, and manuscript final version approval.

### Conflict of Interest

The authors declare that the research was conducted in the absence of any commercial or financial relationships that could be construed as a potential conflict of interest.

## Abbreviations

CRC: colorectal cancer
5-FU: 5-fluouracil
SGLT1: sodium/glucose transporter 1
GLUT: glucose transporter
RIP: receptor-interacting protein kinase
MPC: mitochondrial pyruvate carrier
TCA: tricarboxylic acid
ROS: reactive oxygen species
FBS: fetal bovine serum
BHA: butylated hydroxyanisole
NAC: N-Acetyl L-Cysteine
LDH: lactodehydrogenase
ROS: reactive oxygen species.
