# Supplementary material for: Glucose Metabolites Exert Opposing Roles in Tumor Chemoresistance
Source: Front Oncol. 2019 Nov 21;9:1282. doi: 10.3389/fonc.2019.01282 (PMC6881467; doi:10.3389/fonc.2019.01282)
Supplement: Supplementary file 1 [file Table_1.DOCX]

**Suppl Table 1. Characteristics of patients with CRC and their responses to chemotherapy containing high dose of 5-fluorouracil.** Independent samples *t*-tests or Chi-squared tests were performed for statistical analysis.

|  | **Responsive**  **(N=14)** | **Nonresponsive**  **(N=11)** | **p-value** |
| --- | --- | --- | --- |
| **Age, years, mean (SD)** | 58.7 (12.2) | 55.8 (17.2) | 0.63 |
| **Gender, females (%)** | 9 (64) | 4 (29) | 0.24 |
| **Stage (%)** |  |  | 0.37 |
| **IIIA** | 2 (14) | 1 (9) |  |
| **IIIB** | 12 (86) | 8 (73) |  |
| **IIIC** | 0 (0) | 2 (18) |  |
| **Tumor diameter, cm, mean (SD)** | 4.3 (1.3) | 4.4 (1.2) | 0.85 |
| **Tumor location (%)** |  |  | 0.73 |
| **Colon** | 10 (71) | 6 (55) |  |
| **Sigmoid-Rectum** | 2 (14) | 2 (18) |  |
| **Rectum** | 2 (14) | 3 (27) |  |
