# Supplementary material for: Glucose Metabolites Exert Opposing Roles in Tumor Chemoresistance
Source: Front Oncol. 2019 Nov 21;9:1282. doi: 10.3389/fonc.2019.01282 (PMC6881467; doi:10.3389/fonc.2019.01282)
Supplement: Supplementary file 2 [file Data_Sheet_1.PDF]

SUPPLEMENTARY MATERIALS AND METHODS

Surgical specimens from colorectal cancer patients

Colorectal cancer (CRC) patients undergoing adjuvant chemotherapy after surgery at National Taiwan University Hospital (NTUH) were recruited as subjects. Surgical specimens from age- and tumor stage-matched patients who were responsive (N=14) or nonresponsive (N=11) to adjuvant chemotherapy regimens containing high dose 5-fluorouracil (5-FU) were assessed. Written informed consent was obtained from all study subjects, and approval for this study was granted by the Research Ethics Committee of NTUH (200912049R).

Animal models of CRC

Specific pathogen-free BALB/c mice at the age of 6-8 weeks old were used in this study. The animals were purchased from and raised in temperature-controlled rooms (23 ± 2°C) with 12 h light-dark cycles in the animal facility of National Taiwan University College of Medicine (NTUCM). Mice were fed standard chow and water *ad libitum*. Experimental procedures were approved by the Laboratory Animal Care Committee of NTUCM.

Mice were subjected to protocols for chemical induction of CRC [1; 2]. The mice were injected intraperitoneally (i.p.) with azoxymethane (AOM) (10 mg/kg body weight; Sigma, St. Louis, MO, USA) at the beginning of the experiment (day 0). After 7 d, 2% dextran sodium sulfate (DSS) (Sigma) was administered in the drinking water for 4 d, followed by 3 d of regular water. This cycle of AOM/DSS was performed 3 times. Body weight was measured every week, and the animals were euthanized with pentobarbital overdose on day 84 for macroscopic inspection of tumor numbers and sizes. The tumor samples were fixed and processed for histological staining with hemotoxylin and eosin dye. Expression levels of glucose transporters were determined by RT-PCR and Western blotting.

Xenograft mouse models

The human colorectal adenocarcinoma cell line HT29 (ATCC#HTB-38) was injected into immunodeficient mice to create a xenograft tumor model. The HT29 cells were suspended in a 1:3 ratio of Matrigel (Corning #354248) to culture medium containing normal glucose (5 mM) or high glucose (25 mM). Immunodeficient NOD.CB17-Prkdc(scid)/JNarl mice (NOD/SCID) were used as the recipients to allow xenograft tumor growth. HT29 cells (2 x 10^6) in a 200-µl volume of Matrigel-medium containing saline vehicle and either normal or high glucose were subcutaneously injected into the right and left flanks of each mouse, respectively. In another mouse

group, HT29 cells suspended in the Matrigel-medium containing 5-FU (0.02 mM) and either normal or high glucose were injected in the same manner. The tumor size and body weight were monitored every 2-3 days until the tumor volume reached 2500 mm^3 as the end point. After euthanization with pentobarbital overdose, mouse tumors were excised and the exact size of the tumors was calculated. Moreover, tumors were fixed in 4% paraformaldehyde and processed for H&E staining.

Immunofluorescence staining

Mouse tissue sections were quenched with 50 mM NH_4Cl in PBS, blocked with 2% normal goat serum, and stained with anti-GLUT1, anti-GLUT2, anti-GLUT3, anti-GLUT4, rabbit anti-SGLT1 (1:1000, Millipore, MA), or isotype control antibodies. After washing with PBS, tissues stained for SGLT1 were incubated with biotin-conjugated goat anti-rabbit IgG (1:1000, Molecular Probes, Carlsbad, CA) for one hour, followed with a streptavidin-conjugated Alexa Fluor® 488 fluorescent probe (1:1000, Molecular Probes) for one hour; tissues stained for GLUTs were incubated with goat anti-mouse IgG conjugated to Alexa Fluor® 488 fluorescent probe (1:1000, Molecular Probes) for one hour. All tissues were stained with Hoechst dye to visualize cell nuclei. The slides were mounted with aqueous mounting media and viewed under a Zeiss fluorescence microscope.

Western blotting

Colonic tissues or tumor samples were homogenized in ice-cold complete RIPA buffer, and the lysate was sonicated and centrifuged. The protein concentration of the supernatant was adjusted to 5 mg/ml and diluted at a 1:1 volume ratio in 2× electrophoresis sample buffer containing 2% (w/v) SDS, 100 mM DTT, and 62.5 mM Tris/HCl (pH 6.8). Samples were then heated to 95°C in a heat block for 5 minutes, and stored at –20°C until used for immunoblotting. The extracted proteins were separated by SDS-PAGE, and the resolved proteins were electrotransferred onto membranes. After blocking with 5% non-fat milk in TBS, the membrane was incubated with primary antibodies at 4°C overnight. Membranes were washed with 0.1% Tween 20 in TBS and incubated with either horseradish peroxidase-conjugated secondary antibodies including goat anti-rabbit or anti-mouse IgG (1:1000, Cell Signaling). The antigens were revealed and band density quantified by photoimage analysis.

The primary antibodies included anti-GLUT1, anti-GLUT2, anti-GLUT3, anti-GLUT4, rabbit anti-SGLT1 (1:1000, Millipore, MA). Moreover, monoclonal mouse anti-β-actin (1:10000, Sigma) was also used to control for equal loading in each sample.

Cell culture models

The human colorectal carcinoma cell lines HT29, HCT116, SW480, and Caco-2 were grown in Dulbecco's Modified Eagle's Medium (DMEM) containing 5 mM glucose and no pyruvate (Life Technologies, Carlsbad, CA) [1; 2; 3; 4; 5; 6]. Cells were exposed to various doses of 5-fluorouracil (5-FU) for 48 hours in culture medium containing 1, 5, or 25 mM glucose [1; 3]. In other experiments, cells were challenged with 5-FU in the presence of equimolar concentrations of a cell-permeable pyruvate derivative, ethyl pyruvate, in glucose- and pyruvate-free culture media [3; 4]. Moreover, ATP-encapsulated and empty liposomes at 0-1000 µM were added to 5-FU-treated cells in glucose- and pyruvate-free culture medium [4]. The liposomes were provided by Dr. Chin-Tin Chen from the Department of Biochemical Science and Technology, National Taiwan University [7]. All reagents were purchased from Sigma, except the lipids bought from Avanti Polar Lipids (Birmingham, AL).

In other settings, cells were treated with necrostatin-1 (a RIP1 inhibitor) or ZVAD-FMK (a pan-caspase inhibitor), phloretin (a GLUT inhibitor), phloridzin (a SGLT-1 inhibitor), iodoacetate (an inhibitor to glyceraldehyde-3-phosphoate dehydrogenase), UK5099 (an inhibitor to mitochondrial pyruvate carrier), or antioxidants such as butylated hydroxyanisole (BHA) and N-Acetyl L-Cysteine (NAC), prior to 5-FU exposure in glucose-containing medium. The reagents were purchased from Sigma.

Spheroid cultures

Cells were plated as three-dimensional spheroid cultures based on previous protocols [1; 8] with some modification. The monolayered cells were trypsinized and resuspended in DMEM containing 25 mM glucose. The cells were then mixed with ice-cold Matrigel (Corning #354234) at 3:1 ratio with cell culture medium, and a volume of 300 µl was seeded per well in 24-well plates. After gel polymerization, 300 µl of culture medium was overlaid. Spheroids were cultured for four days and exposed to 5-FU for 48 hours.

Twenty-four hours prior to 5-FU addition, the overlaying medium was changed to DMEM containing 5 mM glucose. The spheroids were then treated with 5-FU by replacing the overlaying medium with cell culture medium (900 µl) containing various doses of glucose and pyruvate, or liposomal ATP. The spheroid size and structure before and after 5-FU exposure were observed, and the microscopic images were captured using a CCD camera, and analyzed using ImageJ software 1.47v. The spheroid size and structure before and after 5-FU exposure were observed, and the microscopic images were captured using a CCD camera, and

analyzed using ImageJ software 1.47v. The percentage of spheroids with structural dissociation was determined. Destructed spheroids were defined as lacking a circular shape and displaying indentation and notches on the spheroid surface. A total number of 100-120 spheroids were quantified per experimental group.

Measurement of ATP, pyruvate, and lactate contents

Cell lysates were measured for the levels of ATP (Invitrogen), pyruvate (Biovision, Milpitas, CA, USA), and lactate (Biovision) by using commercial assay kits in accordance with manufacturer's instruction. The ATP concentrations in liposomes were also quantified using the kit.

Cell viability tests

The cell viability was determined by using a commercialized tetrazolium dye MTT (3-(4,5-dimethylthiazol-2-yl)-2,5-diphenyltetrazolium bromide) assay (Cayman Chemical, Ann Arbor, MI) according to the manufacturer's instruction. The MTT assay is a colorimetric assay for measuring the metabolic activity of NAD(P)H-dependent cellular oxidoreductase enzymes which reflect the number of viable cells present. The half maximum inhibitory concentration (IC50) of 5-FU was calculated using GraphPad Prism software (GraphPad Software Inc., CA, USA).

Measurement of cell death levels

Apoptosis characterized by DNA fragmentation was measured by a Cell Death Detection ELISA kit (Roche, Basel, Switzerland) according to the manufacturer's instruction [1]. Necrocrosis/Necroptosis was measured by lactodehydrogenase (LDH) leakage assay [3; 4]. Briefly, cell culture supernatant was mixed with a reaction mixture of 0.2 mM NADH and 0.36 mM sodium pyruvate in Krebs-Henseleit (K-H) buffer containing 2% bovine serum albumin for spectrophotometric kinetic readings at 340 nm. One unit of LDH activity is defined as the quantity for oxidation of 1 μmol NADH per minutes. Lastly, evaluation of RIP1/3 signaling as a hallmark of necroptosis was assessed by immunoprecipitation of RIP1-RIP3 complex [3]. Briefly, cell lysates were immunoprecipitated with anti-human RIP1 (BD Bioscience, Franklin Lakes, NJ) and incubated with protein G agarose beads followed by heat denaturation. The immune complex was then subjected to reducing SDS-PAGE, and the membranes were incubated with anti-RIP1 (1:1000, BD Bioscience) or anti-RIP3 (1:1000, Abcam, Cambridge, UK) for immunoblotting.

Measurement of mitochondrial free radicals

Mitochondrial-derived reactive oxidative species (ROS) generation was measured

by using MitoSOX (Invitrogen). The cells were incubated with MitoSOX (5 μM) for 20 min after 5-FU treatment, and then subjected to fluoremetric readings [3].

Evaluation of cell cycle progression

Cells treated with 5-FU were harvested for cell cycle analysis by flow cytometry. After the challenge, the cells were washed with PBS and fixed with ice-cold 70% ethanol for 24 h. The cells were incubated with anti-Ki67 antibody (1:500, LSBio) for one hour, followed with goat anti-mouse IgG conjugated to Alexa Fluor® 488 fluorescent probe (1:1000, Molecular Probes). The cells were then incubated with propidium iodide (PI) for 30 min at room temperature. A minimum of 10,000 PI-stained nuclei were analyzed by flow cytometry, and the percentage of cells in the G0, G1, S, and G2–M phases of the cell cycle was determined using FlowJo cell cycle analysis software (FlowJo LLC, Oregon, USA) [1; 2].

Statistical analysis

All values were expressed as mean± SEM, and the means were compared by one-way analysis of variance followed by a Student-Newman-Keuls post hoc test or by Student's *t* test (Sigma Stat). A *P* value less than 0.05 is considered significant.

Study approval

The animal studies were approved by the Laboratory Animal Care Committee of NTUCM. Human sample collection was approved by the Research Ethics Committee of NTUH (200912049R) and written informed consent was received from participants prior to inclusion in the study.

References

[1] W.T. Kuo, T.C. Lee, H.Y. Yang, C.Y. Chen, Y.C. Au, Y.Z. Lu, L.L. Wu, S.C. Wei, Y.H. Ni, B.R. Lin, Y. Chen, Y.H. Tsai, J.T. Kung, F. Sheu, L.W. Lin, and L.C. Yu, LPS receptor subunits have antagonistic roles in epithelial apoptosis and colonic carcinogenesis. Cell Death Differ 22 (2015) 1590-1604.

[2] W.T. Kuo, T.C. Lee, and L.C. Yu, Eritoran Suppresses Colon Cancer by Altering a Functional Balance in Toll-like Receptors That Bind Lipopolysaccharide. Cancer Res 76 (2016) 4684-95.

[3] C.Y. Huang, W.T. Kuo, Y.C. Huang, T.C. Lee, and L.C. Yu, Resistance to hypoxia-induced necroptosis is conferred by glycolytic pyruvate scavenging of mitochondrial superoxide in colorectal cancer cells. Cell Death Dis 4 (2013) e622.

[4] C.Y. Huang, W.T. Kuo, C.Y. Huang, T.C. Lee, C.T. Chen, W.H. Peng, K.S. Lu, C.Y. Yang, and L.C. Yu, Distinct cytoprotective roles of pyruvate and ATP by glucose metabolism on epithelial necroptosis and crypt proliferation in ischaemic gut. J Physiol 595 (2017) 505-521.

[5] L.L. Wu, W.H. Peng, W.T. Kuo, C.Y. Huang, Y.H. Ni, K.S. Lu, J.R. Turner, and L.C. Yu, Commensal Bacterial Endocytosis in Epithelial Cells Is Dependent on Myosin Light Chain Kinase-Activated Brush Border Fanning by Interferon-gamma. Am J Pathol 184 (2014) 2260-2274.

[6] L.L. Wu, H.D. Chiu, W.H. Peng, B.R. Lin, K.S. Lu, Y.Z. Lu, and L.C.H. Yu, Epithelial inducible nitric oxide synthase causes bacterial translocation by impairment of enterocytic tight junctions via intracellular signals of Rho-associated kinase and protein kinase C zeta. Critical Care Medicine 39 (2011) 2087-2098.

[7] P.C. Peng, R.L. Hong, Y.J. Tsai, P.T. Li, T. Tsai, and C.T. Chen, Dual-effect liposomes encapsulated with doxorubicin and chlorin e6 augment the therapeutic effect of tumor treatment. Lasers Surg Med 47 (2015) 77-87.

[8] T. Sato, D.E. Stange, M. Ferrante, R.G. Vries, J.H. Van Es, S. Van den Brink, W.J. Van Houdt, A. Pronk, J. Van Gorp, P.D. Siersema, and H. Clevers, Long-term expansion of epithelial organoids from human colon, adenoma, adenocarcinoma, and Barrett's epithelium. Gastroenterology 141 (2011) 1762-72.
