# Supplementary material for: Glucose Metabolites Exert Opposing Roles in Tumor Chemoresistance
Source: Front Oncol. 2019 Nov 21;9:1282. doi: 10.3389/fonc.2019.01282 (PMC6881467; doi:10.3389/fonc.2019.01282)
Supplement: Supplementary file 3 [file Data_Sheet_2.docx]

**Supplementary Figures**

**Suppl Fig. 1 Glucose uptake by phloretin-sensitive glucose transporters increased cell viability in 5-FU-treated HT29 cells.** HT29 cells were treated with 5-FU (0.01 mM) in culture media containing normal glucose (NG, 5 mM) and high glucose (HG, 25 mM). **(A)** Cell viability by pretreatment with phloretin (PHT, 2.5 mM), phloridzin (PHZ, 2.5 mM), or vehicle (veh) in high glucose conditions. The brackets in each panel indicate the IC50 of 5-FU in HT29 cells. N=6/group. Extra-sum-of-squares *F* tests were performed for IC50 comparison. **(B)** The cellular expression of glucose transporters were examined by Western blots. N=4/group.

**Suppl Fig. 2 The cell viability and LDH leakage of the human colorectal cancer cell lines upon exposure to 5-FU in the presence of 5 and 25 mM of pyruvate.** **(A)** Four human CRC cell lines, including **(a)** HT29, **(b)** HCT116, **(c)** SW480 and **(d)** Caco-2, were treated with 5-FU at various doses for 48 hours in cell media containing 5 or 25 mM of cell-permeable pyruvate derivative, ethyl pyruvate. The cell viability measured by a MTT assay was plotted against the 5-FU doses for calculation of IC50. The brackets in each panel indicate the IC50 of 5-FU in the four cell lines under normal and high pyruvate. Extra-sum-of-squares *F* tests were performed for IC50 comparison. **(B and C)** Presence of high pyruvate (HP, 25 mM) decreased the 5-FU-induced LDH leakage compared to those given normal pyruvate (NP, 5 mM) in glucose-free medium in SW480 and Caco-2 cells. N=6/group. Independent samples *t*-tests were performed. **P*<0.05 *vs.* respective CON; ^#^*P*<0.05 *vs.* respective NP.

**Suppl Fig. 3 Immunoprecitated RIP1/3 complex and mitochondrial free radicals were reduced by pyruvate but not ATP.** HT29, SW480, and Caco-2 cells were exposed to 5-FU in culture media containing empty and ATP-encapsulated liposomes, or normal and high concentrations of pyruvate. **(A)** Addition of liposomal ATP did not inhibit 5-FU-induced RIP1/3 complex formation. **(B)** High pyruvate blocked the RIP1/3 complex formation caused by 5-FU. The relative densitometric values of RIP3 over RIP1 were shown. N=4/group. **(C)** The 5-FU-induced mitochondrial superoxide production was attenuated by high pyruvate in SW480 cells. **(D)** The 5-FU-induced mitochondrial superoxide production was attenuated by high pyruvate in Caco-2 cells. N=6/group. Independent samples *t*-tests were performed. **P*<0.05 *vs.* respective CON; ^#^*P*<0.05 *vs.* 5-FU in Empty or NP.

**Suppl Fig. 4 Pretreatment with antioxidants partly inhibited the cell cycle changes caused by 5-FU.** HT29 cells were pretreated with NAC (10 mM) prior to the exposure of 5-FU. The cell cycles were analyzed by flow cytometry using staining of Ki67 (as FL1) and propidium iodine (PI) (as FL2). **(A)** Dot plot of cell cycles. **(B)** Percentage (%) of cells in each phase. **(C)** Ratio of cells in G1 to G0 phases. Independent samples *t*-tests were performed. **P*<0.05 *vs.* respective CON. ^#^*P*<0.05 *vs.* respective Veh.

**Suppl Fig. 5 High pyruvate attenuated 5-FU-induced G0/G1 shift in SW480 and Caco-2 cells. (A)** SW480 cells and **(B)** Caco-2 cells were exposed to 5-FU in the presence of normal pyruvate (NP, 5 mM) and high pyruvate (HP, 25 mM). The cell cycles were analyzed by flow cytometry using staining of Ki67 (as FL1) and propidium iodide (PI) (as FL2). The panels showed **(a)** Percentage (%) of cells in each phase, and **(b)** Ratio of cells in G1 to G0 phases, under NP and HP. N=4/group. Independent samples *t*-tests were performed. **P*<0.05 *vs.* respective CON; ^#^*P*<0.05 *vs.* respective NP.

**Suppl Fig. 6 Representative images of HT29 spheroid cultures before (day 0) and after (day 2) exposure of 5-FU under normal and high pyruvate conditions *in vitro*.** Bar: 10 μm. N=6/group.
