# Supplementary figures and images for: Glucose Metabolites Exert Opposing Roles in Tumor Chemoresistance
Source: Front Oncol. 2019 Nov 21;9:1282. doi: 10.3389/fonc.2019.01282 (PMC6881467; doi:10.3389/fonc.2019.01282)

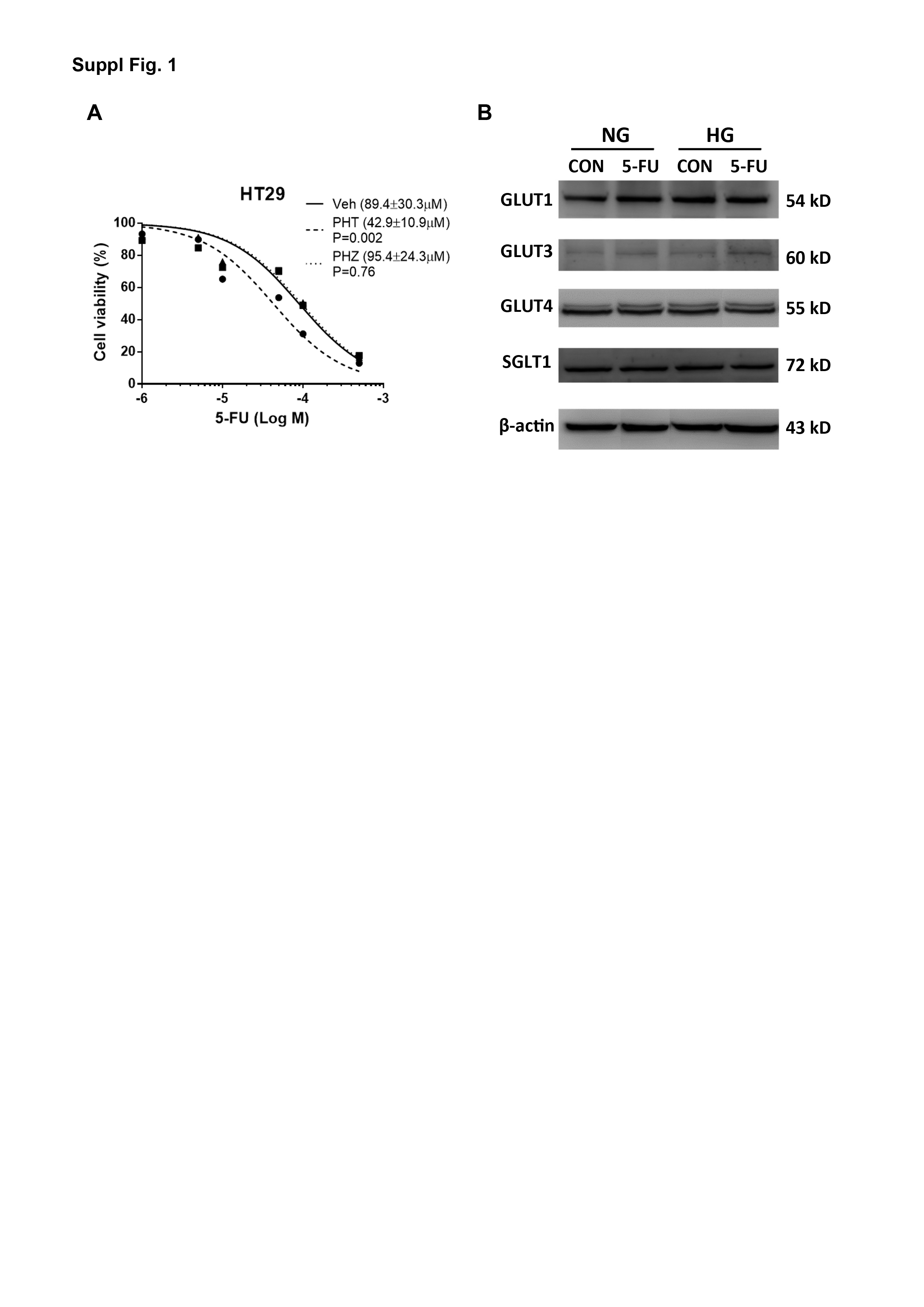

Supplement: Supplementary file 4 [file Image_1.TIF]

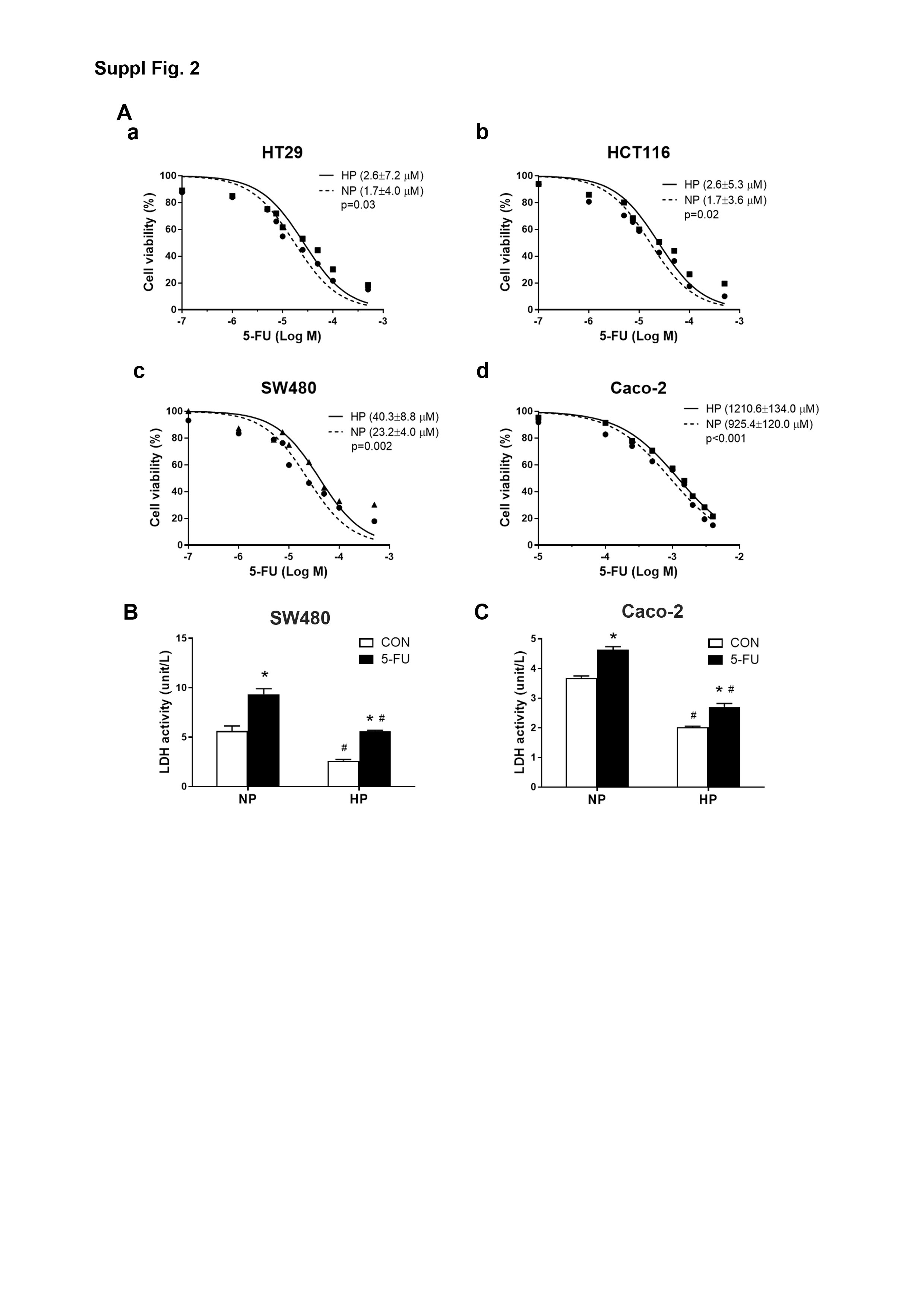

Supplement: Supplementary file 5 [file Image_2.TIF]

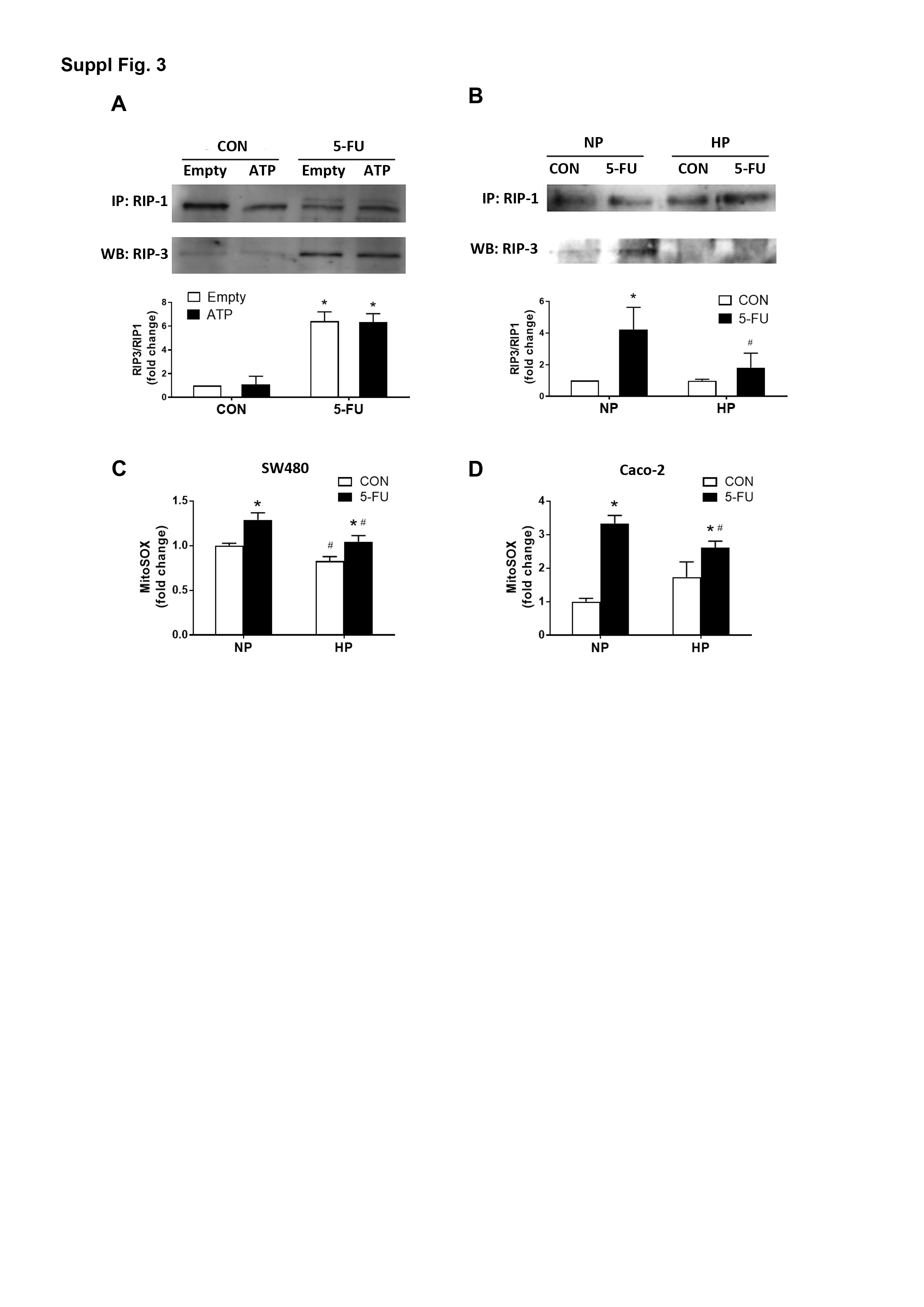

Supplement: Supplementary file 6 [file Image_3.TIF]

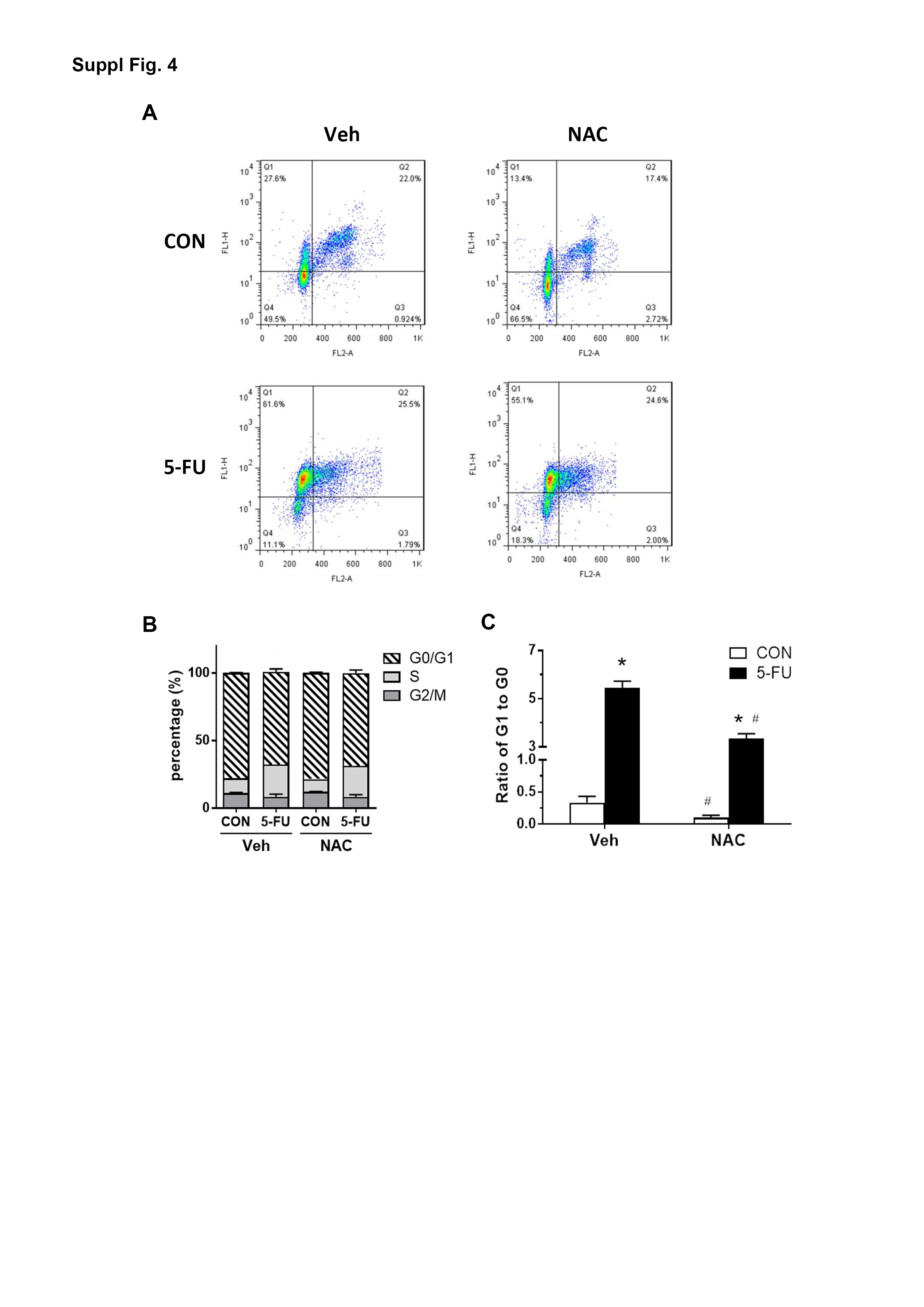

Supplement: Supplementary file 7 [file Image_4.TIF]

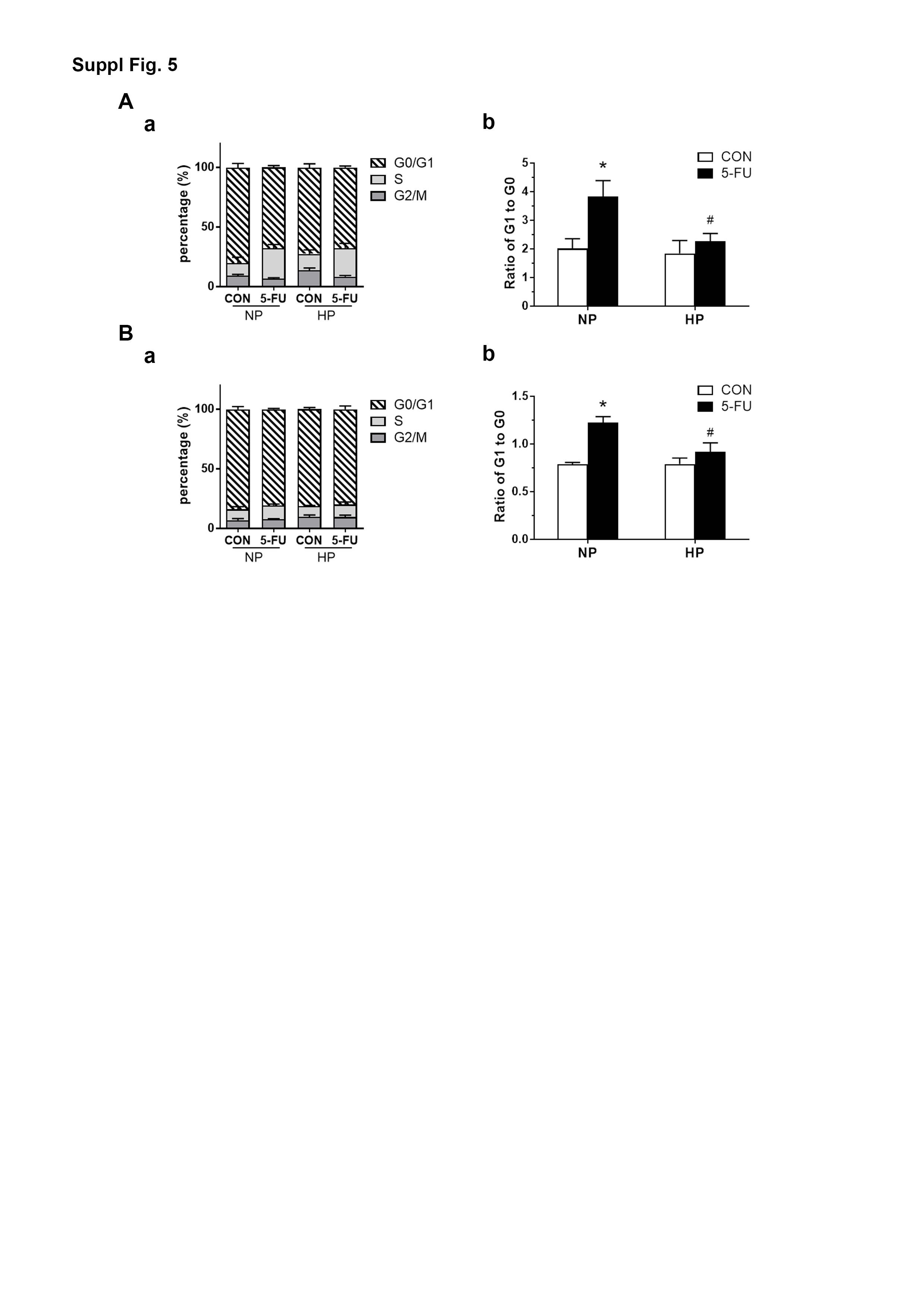

Supplement: Supplementary file 8 [file Image_5.TIF]

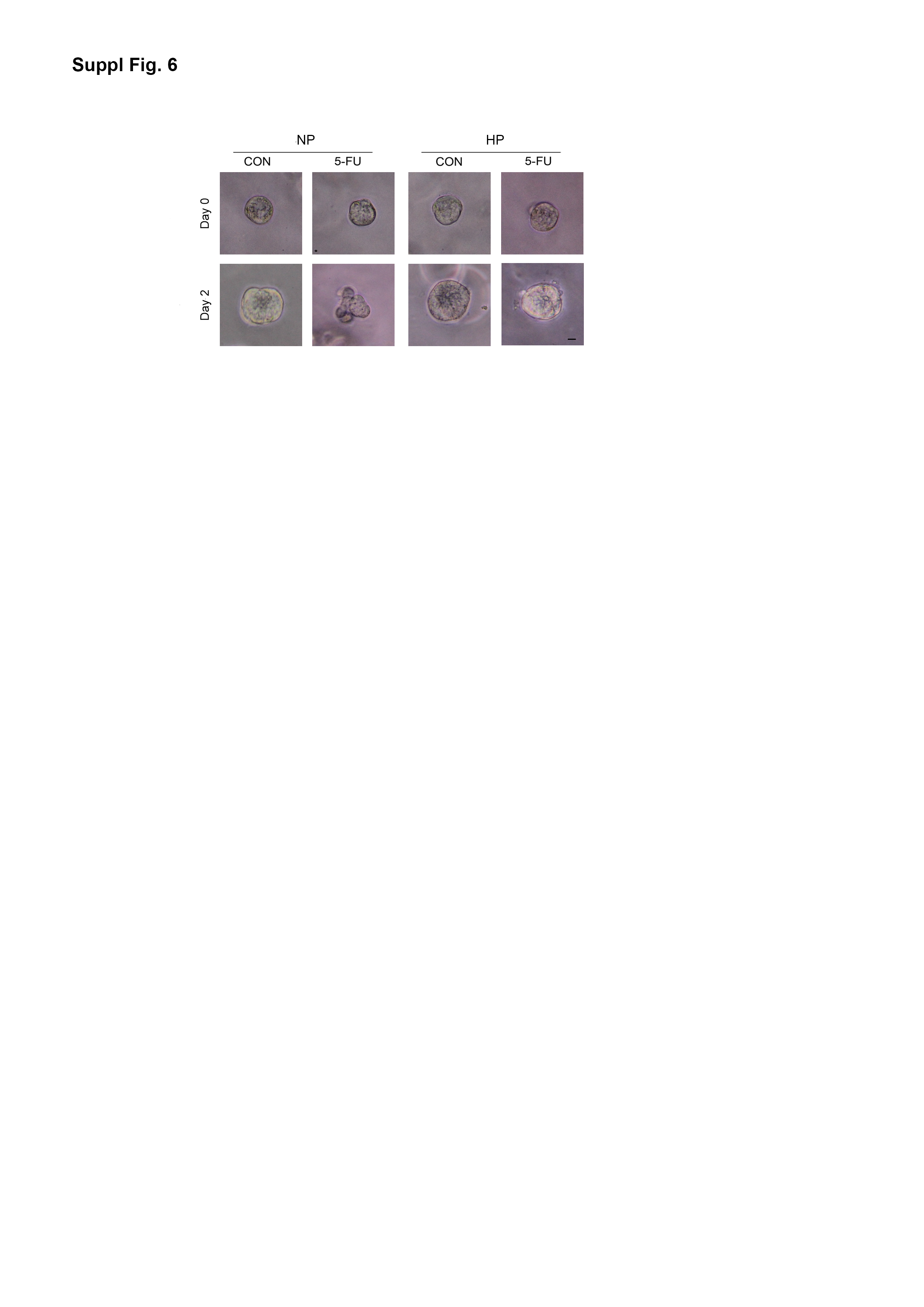

Supplement: Supplementary file 9 [file Image_6.TIF]
